# Tuning Metallic Co_0.85_Se Quantum Dots/Carbon Hollow Polyhedrons with Tertiary Hierarchical Structure for High-Performance Potassium Ion Batteries

**DOI:** 10.1007/s40820-019-0326-5

**Published:** 2019-11-01

**Authors:** Zhiwei Liu, Kun Han, Ping Li, Wei Wang, Donglin He, Qiwei Tan, Leying Wang, Yang Li, Mingli Qin, Xuanhui Qu

**Affiliations:** 1 Beijing Advanced Innovation Center for Materials Genome Engineering, Institute for Advanced Materials and Technology, University of Science and Technology Beijing, 100083 Beijing, People’s Republic of China; 2 Beijing Key Laboratory of Bio-inspired Energy Materials and Devices, School of Space and Environment, Beihang University, 100191 Beijing, People’s Republic of China; 3 Department of Chemical Engineering, Polytechnique Montreal, Montreal, QC H3C 3A7 Canada

**Keywords:** Cobalt selenides, Quantum dots, Potassium-ion batteries, Tertiary hierarchical structure, Hollow dodecahedron

## Abstract

Potassium-ion batteries (KIBs) are a potential candidate to lithium-ion batteries (LIBs) but possess unsatisfactory capacity and rate properties. Herein, the metallic cobalt selenide quantum dots (Co_0.85_Se-QDs) encapsulated in mesoporous carbon matrix were designed via a direct hydrothermal method. Specifically, the cobalt selenide/carbon composite (Co_0.85_Se-QDs/C) possesses tertiary hierarchical structure, which is the primary quantum dots, the secondary petals flake, and the tertiary hollow micropolyhedron framework. Co_0.85_Se-QDs are homogenously embedded into the carbon petals flake, which constitute the hollow polyhedral framework. This unique structure can take the advantages of both nanoscale and microscale features: Co_0.85_Se-QDs can expand in a multidimensional and ductile carbon matrix and reduce the *K*-intercalation stress in particle dimensions; the micropetals can restrain the agglomeration of active materials and promote the transportation of potassium ion and electron. In addition, the hollow carbon framework buffers volume expansion, maintains the structural integrity, and increases the electronic conductivity. Benefiting from this tertiary hierarchical structure, outstanding *K*-storage performance (402 mAh g^−1^ after 100 cycles at 50 mA g^−1^) is obtained when Co_0.85_Se-QDs/C is used as KIBs anode. More importantly, the selenization process in this work is newly reported and can be generally extended to prepare other quantum dots encapsulated in edge-limited frameworks for excellent energy storage.

**Electronic supplementary material:**

The online version of this article (10.1007/s40820-019-0326-5) contains supplementary material, which is available to authorized users.

## Highlights

- Metallic cobalt selenide quantum dots encapsulated in mesoporous carbon matrix were prepared via a direct hydrothermal method.
- The cobalt selenide/carbon composite (Co_0.85_Se-QDs/C) possesses tertiary hierarchical structure, which is the primary quantum dots, the secondary petals flake, and the tertiary hollow micropolyhedron framework.
- Benefiting from this tertiary hierarchical structure, the Co_0.85_Se-QDs/C electrode as potassium-ion batteries anode shows an outstanding *K*-storage performance.

**Electronic supplementary material:**

The online version of this article (10.1007/s40820-019-0326-5) contains supplementary material, which is available to authorized users.

## Graphical abstract

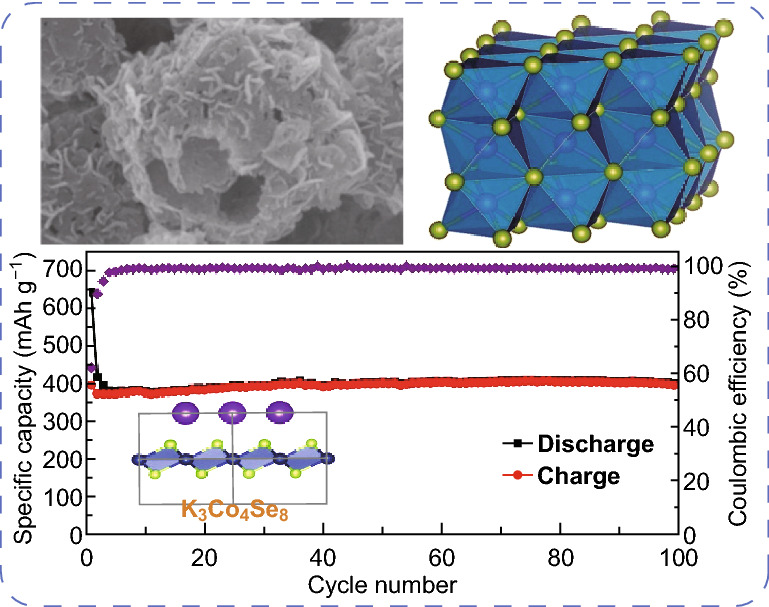

## Introduction

Rapidly increasing demands for energy on a global scale give impetus to the application and development of new energy. Lithium-ion batteries (LIBs) as a promising secondary battery have been extensively used in the fields of vehicles and electronic devices due to their high capacity [1–3]. However, the rising cost and limited reserve of Li resources restrict the further practical applications of LIBs, which offers the potential opportunities for other rechargeable batteries, such as aluminum-ion, potassium-ion, sodium-ion, magnesium-ion, and dual-ion batteries [4–11]. Owing to the substantial reserves of potassium and sodium elements on earth, potassium-ion batteries (KIBs) and sodium-ion batteries (NIBs) have been widely studied in energy storage fields. But the commercial graphite could not be used as NIBs anode, and the reduction potential of Na is − 2.71 V (vs. SHE), lower than − 3.04 V (vs. SHE) of Li [12]. However, the reduction potential of K is − 2.93 V (vs. SHE), very close to that of Li, which offers a new thought for the next generation of energy storage battery systems [13–15]. Compared with NIBs, the heavy K ions may be not very competitive due to the increase in weight; however, the total mass of the KIBs is not vital for large-scale energy storage because the energy density is low priority [14].

In recent years, the KIBs anode materials are mainly graphite, graphene, soft/hard carbon, tin-based composites, transition-metal oxides, sulfides, and phosphides [16–23]. Nevertheless, the low cycling capacity and rate capability make the researchers seek new anode materials. Many researchers focus on the transition-metal chalcogenide (sulfide, selenide, and telluride) due to the higher capacity than conventional intercalation electrode materials. Among them, metal selenides used as KIBs anodes possess an increased attention on account of their good thermal stabilities and favorable mechanical and electrical conductivities [24, 25], which make them to obtain better rate performance and electrochemical stability. Recently, cobalt selenides with different nanostructures have been reported and exhibit excellent performance in the energy storage fields [26]. Nevertheless, the fast capacity fading resulting from the structural failure in the initial several cycles is still the bottleneck for its practical application, so it is imperative to use carbon matrix to buffer the volume expansion of cobalt selenide nanocomposites in the discharge/charge cycle.

Moreover, the miniaturization and dispersion of active materials on carbon support are also difficult issues to be solved. Although the active materials with tiny size [e.g., quantum dots (QDs)] have the ability to expand in a ductile and multidimensional surrounding [27], small-sized nanoparticles are prone to agglomeration and form inactive clusters, and thus improving the dispersion of active materials on carbon matrix is imperative [28]. Meanwhile, constructing 3D hierarchical network can profitably enhance the structural integrity and their electrochemical properties. The 3D hierarchical structure can effectively accommodate the mechanical stress and rapidly transfer ions, improving the rate performance and reducing the polarization. Therefore, controlling the uniform dispersion of nanoparticles in hierarchical structure becomes the focus of researches. Generally, the metal organic frameworks (MOFs) structure can meet the above requirements [29–31]. QDs, carbon matrix, and hierarchical structure can be obtained by further processing different MOFs. Lou et al. reported that the hierarchical structure of CoSe@carbon nanoboxes was synthesized through the template-engaged reaction [32], which manifests excellent electrochemical performance for LIBs. However, the conventional selenization process of the MOFs consumes a mass of energy and produces harmful gases. Hydrothermal method is simple to carry out at a lower temperature and reduces energy consumption. So far, no research was reported about the one-step modification of MOFs by hydrothermal selenization.

In this work, we prepare the cobalt selenide quantum dots (Co_0.85_Se-QDs) encapsulated in mesoporous polyhedral carbon (C) matrix through one-step hydrothermal selenization for the first time. The shape-controllable Co_0.85_Se-QDs/C composite with petal-shaped shell exhibits good specific surface area and effectively buffers relative volume change, which provides outstanding specific capacity, excellent rate performance, and favorable cycling stability. Thus, the Co_0.85_Se-QDs/C electrode shows an ultrahigh capacity of 402 mAh g^−1^ after 100 cycles at the current density of 50 mA g^−1^ with a good capacity retention of 99%. Besides, the reduction potential of the Co_0.85_Se-QDs/C electrode (0.5 V) overtops the plating voltage of K metal (0.01 V), which can avoid the formation of metal dendrites and enhance their safety. The phase transition process was further investigated by the first-principle calculations.

## Experimental

### Synthesis of Co-Based Polyhedron Precursor

ZIF-67 polyhedron (denoted as Co polyhedron) precursor was synthesized on the basis of a previously reported literature with slight modification [33]. Cobalt nitrate hexahydrate powder (0.29 g) was added into methanol solution (25 mL) and kept stirring in a beaker for 10 min to form a pink solution. Afterward, a mixture of 2-methylimidazole (0.328 g) and methanol solution (25 mL) was dropwise added into the former beaker under continuous stirring. When two solutions were mixed together, stirring was stopped. Finally, the purple precipitates were collected by centrifugation after aging at room temperature for 24 h, washed three times with absolute ethyl alcohol, and dried in a vacuum oven for further use.

### Synthesis of Co_0.85_Se-QDs/C Composite

In a typical route, Co polyhedron precursor (0.039 g), H_2_SeO_3_ (0.013–0.052 g), and hydrazine hydrate (N_2_H_4_·H_2_O) (0–20 mL) were mixed to form a mixture solution. Then, the mixture solution was transferred into the Teflon-lined stainless-steel autoclaves and ultrasonically stirred for 15 min. The autoclave was sealed and retained at 140–220 °C for 24 h in a drying oven without any agitation and then allowed to gradually cool down to room temperature after heat treatment. The final sample was obtained by free sedimentation, washed with absolute ethyl alcohol, and dried in a vacuum box at 65 °C for 4 h. Based on different amounts of hydrazine hydrate (20, 10, 2, and 0 mL), the as-prepared samples are named as Co_0.85_Se-QDs/C-20, Co_0.85_Se-QDs/C-10, Co_0.85_Se-QDs/C-2, and Co_0.85_Se-QDs/C-0, respectively.

### Materials Characterizations

The phases of all the materials were measured by the X-ray powder diffraction (XRD) with Cu Kα radiation (Rigaku D/max-RB12 X-ray diffractometer). The morphologies and surface elemental compositions of the samples were observed by field emission scanning electron microscopy (FESEM, JEOL, JSM-7001F). The transmission electron microscopy (TEM) and high-resolution transmission electron microscopy (HRTEM) images were observed by the transmission electron microscopy (JEOL, JEM-2010). The energy-dispersive X-ray spectra (EDS) and scanning transmission electron microscopy (STEM) analyses were implemented by using a Hitachi HD2700C (200 kV). X-ray photoelectron spectra (XPS) were carried out with an ESCALAB 250 spectrometer (PerkinElmer) to explore the valence states of surface elements. The Raman spectra (Renishaw RM2000) with a wavelength of 532 nm were used to investigate the crystallization degree of carbon at room temperature. Differential scanning calorimetry (DSC) and thermogravimetry (TG) were tested using Seiko 6300 instrument. Brunauer–Emmett–Teller (BET) method was used to characterize the specific surface area of the samples.

### Electrochemical Measurements

Electrochemical measurements were carried out using the CR2023-type coin cells. The working electrode was the as-prepared Co_0.85_Se-QDs/C, and the K metal worked as both the reference and counter electrodes. The synthesized Co_0.85_Se-QDs/C were mixed with polyvinylidene fluoride (PVDF) in the weight ratio of 8:2. The coated foil was punched into pellets with a diameter of 10 mm and the mass loading of ~ 1.2 mg cm^−2^. A nonaqueous electrolyte solution consisted of 1 M KFSI and dimethyl ether (DME). The separator is the glass fiber (GF/D) from Whatman. The coin cells were assembled and disassembled in an argon atmosphere glove box. The coin cells were galvanostatically discharged and charged using LAND-CT2011A battery-testing instrument under 25 °C, and the testing voltage performed ranges from 0.01 to 2.5 V. Cyclic voltammograms (CVs) scanned at 0.1 mV s^−1^ in a voltage window of 0.01–2.5 V were measured by an electrochemistry workstation (CHI660E).

### Computational Section

The first-principle calculations were carried out using density functional theory (DFT) with the Perdew–Burke–Ernzerhof (PBE) form of generalized gradient approximation functional (GGA) [34]. The Vienna Ab initio Simulation Package (VASP) was employed [35–38]. The plane-wave energy cutoff was set as 400 eV. The SCF energy (converged to 1.0 × 10^−6^ eV atom^−1^) and the Hellman–Feynman force (converged to 0.01 eV Å^−1^) were set as the convergence criterion for geometry optimization. We chose the Co_7_Se_8_ closest to Co_0.85_Se as the initial structure for K-doped/replacement reaction. The first Brillouin zone was sampled in the Monkhorst–Pack grid [39] with the 4 × 4×4 k-point mesh for the initial structures of Co_7_Se_8_ with 15 atoms for the potassium replacement reaction, and the same dense mesh for the reaction products (K_*x*_Co_(7-*x*)_Se_8_).

As potassium reacted with the Co_7_Se_8_, refer to reaction (1):

1 $${\text{Co}}_{7} {\text{Se}}_{8} + x{\text{K }} \to {\text{ K}}_{x} {\text{Co}}_{(7 - x)} {\text{Se}}_{8} + x{\text{Co}}$$

Thus, the formation energy for reaction (1) is defined as:

2 $$E_{\text{f}} = E_{\text{tot}} ({\text{K}}_{x} {\text{Co}}_{(7 - x)} {\text{Se}}_{8} ) \, + xE({\text{Co}}) \, {-}xE({\text{K}}) \, - E_{\text{tot}} ({\text{Co}}_{7} {\text{Se}}_{8} )$$

3 $$E({\text{Co}}) \, = \, 1/2\;E_{\text{tot}} ({\text{Co}})$$

4 $$E({\text{K}}) \, = \, 1/2\;E_{\text{tot}} ({\text{K}})$$

where *E*_tot_ is the total energy of corresponding compounds and *E*_tot_ (K) and *E*_tot_ (Co) are the total energies of bulk K and Co, respectively. *x* is the number of Co atoms replaced by K atoms. The negative (positive) *E*_f_ denotes the K replacement can be easily (hardly) occurred.

## Results and Discussion

**Fig. 1:**
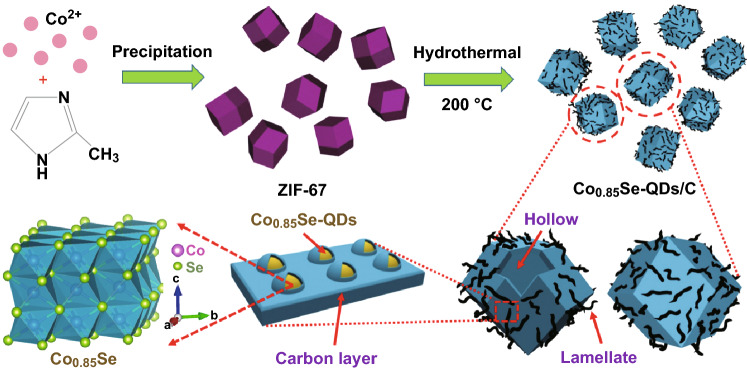
Schematic illustration of the synthesis process for Co_0.85_Se-QDs/C composite

The prepared process of the hierarchical Co_0.85_Se-QDs/C composite materials is schematically shown in Fig. 1. Firstly, the positively charged Co^2+^ and 2-methylimidazole aqueous solution were added to allow the heterogeneous nucleation and in situ growth of Co-based zeolitic imidazolate framework (ZIF-67) crystal. The subsequent step was the selenization process by hydrothermal synthesis using hydrazine hydrate and selenious acid as reductant and selenium source, respectively. During the hydrothermal process, the ZIF-67 as a precursor reacted with selenide ion to form cobalt sulfide QDs accompanied with the formation of mesoporous carbon polyhedra via the carbonization of the organic ligands. As a result, tertiary hierarchical Co_0.85_Se-QDs/C composite was obtained.

**Fig. 2:**
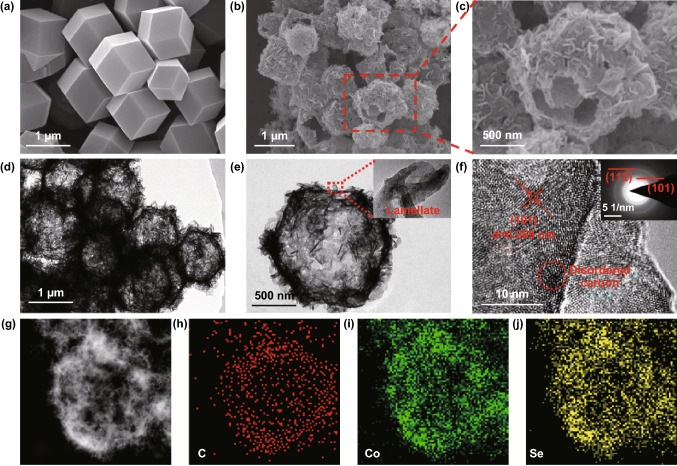
**a** SEM images of Co-based precursor. **b, c** SEM images and **d, e** TEM images of Co_0.85_Se-QDs/C-20. **f** High-resolution TEM image and SAED pattern of Co_0.85_Se-QDs/C-20. **g** STEM image of Co_0.85_Se-QDs/C-20 and **h**–**j** the corresponding elemental mappings of the C, Co, and Se elements

The FESEM and TEM images of ZIF-67 show that the precursor consists of uniform polyhedral particles with a size of ~ 1 um (Figs. 2a and S1). The XRD pattern of Co polyhedron precursor exhibits typical ZIF-67 characteristic (Fig. S2a). The FESEM images (Fig. 2b, c) show that Co_0.85_Se-QDs/C-20 composite has small divergent lamellas on the surface of hollow polyhedron. The TEM images (Fig. 2d, e) further demonstrate the hollow polyhedral structure with petal-shaped shell, which is in good agreement with the FESEM observations in Fig. 2c. H_2_SeO_3_ was reduced to Se and further transformed into selenide ion by hydrazine hydrate during the selenization process, reacting with Co polyhedron precursor to form Co_0.85_Se due to its thermodynamic stability and lower solubility [40]. The formation of hollow structure comes from the diffusion of Co^2+^ from interior to surface and exceeds the diffusion of Se^2−^, which can be considered as the Kirkendall effect [41, 42].The lamellate shell (insert of Fig. 2e) is attributed to the structural collapse of Co precursor during the selenide reaction [43]. The lattice fringe of Co_0.85_Se-QDs in magnification could be clearly observed with the size of 0.269 nm (Fig. 2f), which coincides with the (101) plane of Co_0.85_Se. The Co_0.85_Se-QDs/C-20 composite consists of tertiary hierarchical structure, which is the primary Co_0.85_Se QDs (Fig. S2b), the secondary carbon petal flake, and the tertiary hollow carbon polyhedron framework. The selected area electron diffraction (SAED) image (insert of Fig. 2f) confirms the polycrystalline crystal structure of Co_0.85_Se-QDs. Additionally, the elemental mapping by EDS testing (Fig. 2g–j) verifies the uniform distribution of the carbon, cobalt, and selenium in the Co_0.85_Se-QDs/C-20 composite.

To study the structural evolution and optimize the electrochemical performance, the morphology and particles size of Co_0.85_Se-QDs/C composite are changed by varying the preparation temperature in the selenization process. The as-prepared Co_0.85_Se-QDs/C samples are denoted as Co_0.85_Se-QDs/C-160, Co_0.85_Se-QDs/C-180, and Co_0.85_Se-QDs/C-220 synthesized under the temperatures of 160 °C, 180 °C, and 220 °C, respectively (Fig. S3). When the hydrothermal temperature is 160 °C, the Co_0.85_Se-QDs/C-160 still retains the partial unreacted ZIF-67 polyhedron with broadened XRD peaks due to the incomplete reaction (Figs. S3a, b and S4). With the temperature increasing to 180 °C, the surface of Co_0.85_Se-QDs/C-180 gradually becomes petal-shaped shell (Fig. S3c, d), and XRD intensity of Co_0.85_Se phase is significantly increased and the diffraction peaks of CoSe_2_ phase are reduced. However, Co_0.85_Se-QDs/C-220 polyhedral particles are broken up when the hydrothermal temperature reaches 220 °C (Fig. S3e, f) and the diffraction peaks of Co_0.85_Se, Co_9_Se_8_, and CoSe_2_ phase simultaneously appear in the XRD pattern. Thus, the optimized hydrothermal temperature of 200 °C is chosen in this work.

The amount of selenite also has a significant effect on the phase and morphology of Co_0.85_Se-QDs/C composite. According to the additive amount of selenite from 0.026, 0.039, to 0.052 g, the obtained Co_0.85_Se-QDs/C composites are denoted as Co_0.85_Se-QDs/C-26, Co_0.85_Se-QDs/C-39, and Co_0.85_Se-QDs/C-52, respectively. With the increase in H_2_SeO_3_, the morphology of Co_0.85_Se-QDs/C composite varies from nanosheets to porous spheres (Fig. S5). The diffraction peaks of Co_0.85_Se are gradually weakened, whereas CoSe_2_ phase is gradually strengthened in the XRD pattern (Fig. S6). This result indicates that different amounts of H_2_SeO_3_ in the hydrothermal selenization process can regulate the polyhedral morphology of the products.

To further investigate the growth mechanism of Co_0.85_Se-QDs/C-20, the reductant is controlled to explain the structural evolution from Co-based polyhedron precursor to mesoporous and hollow Co_0.85_Se-QDs/C-20 composite (Figs. S7–S9). When the addition amount of hydrazine hydrate is 10 mL, Co_0.85_Se-QDs/C-10 presents spherical morphology with rough surface (Fig. S7a). The Co_0.85_Se-QDs/C-10 exhibits similar hollow structure to Co_0.85_Se-QDs/C-20 (Fig. S7b). The HRTEM image of Co_0.85_Se-QDs/C-10 shows two different lattice fringes with spacings of 0.269 and 0.238 nm, corresponding to the (101) plane of Co_0.85_Se and the (211) plane of CoSe_2_, respectively (Fig. S7c). The SAED image further verifies the coexistence of Co_0.85_Se and CoSe_2_ in Co_0.85_Se-QDs/C-10, which is in accordance with the XRD results (Fig. S10). When the addition amount of hydrazine hydrate decreases to 2 mL, the Co_0.85_Se-QDs/C-2 shows flake morphology decorated with agglomerated nanoparticles (Fig. S8a–c), and the HRTEM images display that Co_0.85_Se-QDs/C-2 contains monocrystalline Co(OH)_2_ phase and Co_0.85_Se phase (Fig. S8d, e). The SAED image (Fig. S8f) further shows the monocrystalline characteristic of Co(OH)_2_. Without the addition of hydrazine hydrate, the Co_0.85_Se-QDs/C-0 is composed of Co_3_O_4_, Co_0.85_Se, CoSe_2_, Co_9_Se_8_, Co(OH)_2_, and Se phases, which presents the dominating rod-like polycrystal of Co_3_O_4_ (Fig. S9). Using optimized ratio of reducing agent and oxidizer after selenization, pure-phased cobalt selenide with polyhedral structure can be obtained [44, 45]. When the amount of hydrazine hydrate is abundant, selenium and cobalt ions react to form a thin barrier layer of cobalt selenide in the polyhedron surface. The mechanism involved for the hollow architecture is attributed to the ion exchange reaction. As the amount of hydrazine hydrate decreases, only partial selenium ions are formed, and cobalt ions react with hydroxide to form flake cobalt hydroxide. The hydrothermal reaction completely destroys the polyhedron due to the direct effect of selenite to Co-based polyhedron precursor when hydrazine hydrate is not added, leading to the appearance of cobalt oxide nanowires.

**Fig. 3:**
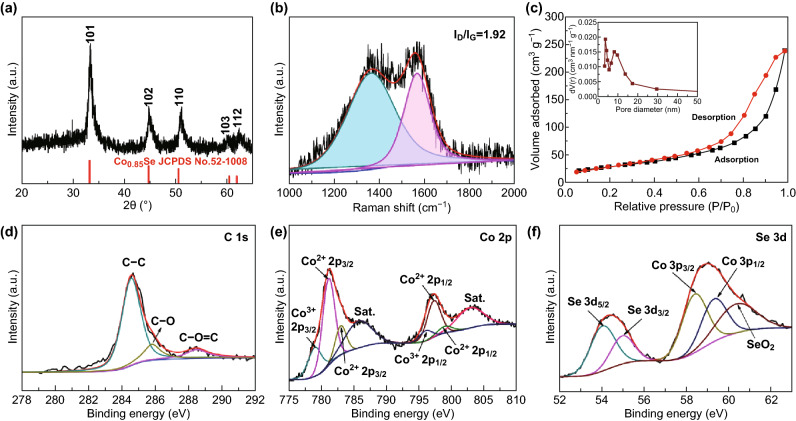
Structural characterizations of the as-synthesized Co_0.85_Se-QDs/C-20. **a** XRD pattern. **b** Raman pattern. **c** N_2_ adsorption isotherm and pore-size distribution. Peak deconvolutions of the XPS spectra: **d** C 1*s*, **e** Co 2*p*, **f** Se 3*d*

Figure 3a reveals the XRD curves of the as-synthesized Co_0.85_Se-QDs/C-20 at 200 °C for 24 h. All the strong diffraction peaks of the sample are assigned to the Co_0.85_Se phase with a hexagonal crystal structure (JCPDS 52-1008), which indicates the complete conversion of ZIF-67 to Co_0.85_Se-QDs/C-20. Moreover, the wide diffraction peaks of the Co_0.85_Se phase are related to large lattice strain and the ultrafine crystalline size [46], which further explains that amorphous carbon shells can prevent Co_0.85_Se nanoparticles from continuously growing in the hydrothermal process, leading to the formation of Co_0.85_Se QDs [47]. No carbon reflection in the XRD pattern is observed due to its amorphous nature. The chemical composition of Co_0.85_Se-QDs/C-20 is further detected by EDS spectrum (Fig. S11), confirming the presence of carbon, cobalt, and selenium elements. The element content of Co_0.85_Se-QDs/C-20 is summarized in Table S1. Figure 3b shows the Raman spectrum of the as-synthesized Co_0.85_Se-QDs/C-20. It can be clearly seen that the intensity of D-band around 1350 cm^−1^ (corresponded to disorder or defective carbon) is much stronger than that of the G-band around 1580 cm^−1^ (corresponded to graphitic carbon). And the relative intensity ratio of D–G-band (*I*_D_/*I*_G_) is 1.92, manifesting its low degree of graphitization. The diverse porosity of the Co_0.85_Se-QDs/C-20 is investigated by N_2_ adsorption/desorption tests (Fig. 3c). The Co_0.85_Se-QDs/C-20 presents a typical type IV curve, indicating the existence of mesoporous structure. The Brunauer–Emmett–Teller (BET) specific surface area of Co_0.85_Se-QDs/C-20 is 112 m^2^ g^−1^. The average pore-size distribution of Co_0.85_Se-QDs/C-20 (insert of Fig. 3c) shows massive mesoporous structure in the range of 4–9 nm. TG–DSC curves of Co_0.85_Se-QDs/C-20 are plotted at 20–800 °C in air (Fig. S12). Based on the TG analysis, the carbon content in the composites is estimated to be approximately 15 wt%.

XPS analysis was performed to confirm the surface electronic state and the chemical composition of Co_0.85_Se-QDs/C-20 (Fig. 3d–f). The survey scan spectrum shows that the elemental components of the Co_0.85_Se-QDs/C-20 are mainly cobalt, carbon, oxygen, and selenium (Fig. S13). The spectra are calibrated by the standard C 1*s* signal located at 284.8 eV. As shown in Fig. 3c, the detected C peaks of C 1*s* spectrum are divided into three peaks, corresponding to the C–C, C–O, and O–C = O bonds located at 284.6, 285.8, and 288.6 eV, respectively. The Co 2*p* spectrum is deconvoluted into four main peaks at the binding energies of 778.5, 780.8, 796.3, and 797.5 eV, corresponding to the Co-Se and Co–O of Co 2*p*_3/2_, Co–Co, and Co–O of Co 2*p*_1/2_, respectively. Besides, two small peaks of Co^2+^ centered at 782.6 and 798.8 eV can correspond to cobalt hydroxides. Furthermore, two shake-up satellites for a higher-energy Co 2*p* signal correspond to the antibonding orbital between selenium and cobalt atoms [48]. Among them, the weak Co–Co bonding of Co 2*p*_1/2_ stems from trace amounts of unreacted Co nanospheres during selenization, and the Co–O bonding could be attributed to the tiny amount of SeO_2_ generated during selenization and partial surface oxidation in air. As exhibited in Fig. 3f, the peaks at 55.5 and 54.6 eV could be assigned to Se 3*d*_3/2_ and Se 3*d*_5/2_, respectively, corresponding to the metal–Se interactions of the Co_0.85_Se-QDs/C-20. The peaks at 58–61 eV are ascribed to oxygen–selenium bonding existing in the sample surface and Co 3p [49]. The d-electron configuration of metal cations can effectively influence the physical characteristics of the metal dichalcogenides [50]. The Co 3*d* electrons of Co_0.85_Se make it to be viewed as a metallic conductor by adopting a low-spin configuration in the form of *t*_2g_^6^*e*_g1_ [51]. The metallic property can benefit the transportation of electrons through active electrodes, leading to an excellent rate performance.

**Fig. 4:**
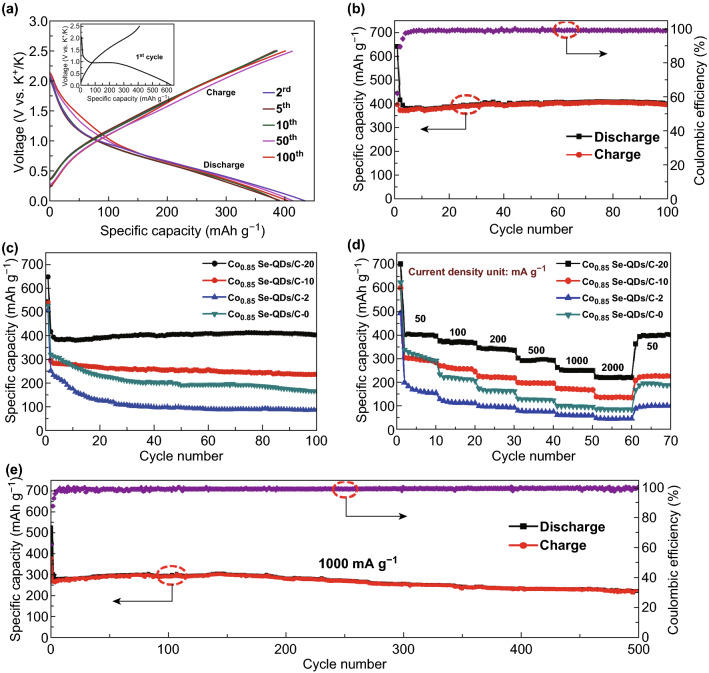
Electrochemical potassium storage performance of Co_0.85_Se-QDs/C composite. **a** The second, third, fifth, tenth, fiftieth, and hundredth charge/discharge profiles of Co_0.85_Se-QDs/C-20 at 50 mA g^−1^ for KIBs (inset is the charge/discharge profiles of the first cycle). **b** Charge/discharge capacity and Coulombic efficiency of Co_0.85_Se-QDs/C-20 at 50 mA g^−1^. Comparison between **c** cycling stability and **d** rate performance under different current densities of the as-prepared Co_0.85_Se-QDs/C samples. **e** Long-term cycling stability and Coulombic efficiency at a high current density of 1 A g^−1^ over 500 cycles

Electrochemical tests were used to evaluate the potassium storage performance of the Co_0.85_Se-QDs/C-20 electrode using the CR2032-type coin cells at 25 °C. The galvanostatic discharge/charge profiles of the Co_0.85_Se-QDs/C-20 electrode at 50 mA g^−1^ are presented in Fig. 4a. The initial discharge/charge capacities are 648 and 401 mAh g^−1^, respectively, which accord with the Columbic efficiency (CE) of 61.8%. The observed capacity loss is due to the formation of the solid electrolyte interphase (SEI) layer on the surface of the electrodes [52], corresponding to the cycle voltammetry (CV) curves of the Co_0.85_Se-QDs/C-20 (Fig. S14). A plateau at around 0.9 V in the first discharge curve can be ascribed to the phase transformation from Co_0.85_Se to Co and K_2_Se [53], and a plateau at around 1.5 V in the first charge curve could be assigned to the inverse phase transformation. After several cycles, the capacity loss of the Co_0.85_Se-QDs/C-20 is nearly negligible, indicating a high discharge/charge reversibility. Meanwhile, the voltage plateau of the Co_0.85_Se-QDs/C-20 (0.5 V) is higher than the plating voltage of potassium metal (0.01 V), which can avoid the risk of dendrite formation.

The cycle performance of the Co_0.85_Se-QDs/C-20 at 50 mA g^−1^ over 100 cycles is shown in Fig. 4b. During the initial cycle, the serious capacity fading can be mainly ascribed to the incomplete formation of SEI layer, which is consistent with the discharge/charge curves. After several cycles of cell activation, the slight capacity rise is ascribed to the activation process during reversible cycle process [54]. Moreover, a high capacity of 402 mAh g^−1^ with a relatively high efficiency around 99% is achieved even after 100 cycles, indicating the excellent cycling stability of Co_0.85_Se-QDs/C-20. Such high capacity is closely related to the hierarchical structure that is advantageous to potassium storage. The primary Co_0.85_Se QDs can fully react with potassium ions and maximize discharge/charge capacity for KIBs. The secondary carbon petal provides stable transportation path for potassium ion and electron, which is favorable for potassiation/depotassiation. The tertiary hollow carbon polyhedron framework prevents the agglomeration of active materials, maintains structural integrity, and boosts the electronic conductivity.

For comparison, the cycling stabilities of Co_0.85_Se-QDs/C-10, Co_0.85_Se-QDs/C-2, and Co_0.85_Se-QDs/C-0 are also tested (Fig. 4c). They present a lower capacity than that of the Co_0.85_Se-QDs/C-20 under the same testing condition. After 100 cycles, Co_0.85_Se-QDs/C-10, Co_0.85_Se-QDs/C-2, and Co_0.85_Se-QDs/C-0 only achieve reversible capacities of 236, 166, and 87 mAh g^−1^, respectively, indicating poor electrochemical performance. The phenomenon could be attributed to the change of phase and structure influenced by the amount of hydrazine hydrate, which produces the side reactions and unstable SEI, causing the structural damage and capacity fading in the discharge/charge process. In addition, the capacity of the Co_0.85_Se-QDs/C-20 is higher than that of other reported selenide anode materials of KIBs (Table S2), demonstrating promising potential for application. Meanwhile, in order to investigate the influence of the crucial role of the electrolytes on potassium storage performance, we compare the cycling stability of Co_0.85_Se-QDs/C-20 in a voltage range of 0.01–2.5 V at 50 mA g^−1^ with selected four electrolytes of 0.8 M KPF_6_ in EC/DEC and 1 M KPF_6_ in diglyme (Figs. S15, S16). The Co_0.85_Se-QDs/C-20 composite shows high initial capacities of 714 and 736 mAh g^−1^, respectively, but the rapid capacity fading after ten cycles is ascribed to side reaction in the discharge/charge process. The cycling performance of Co_0.85_Se-QDs/C-20 electrode which used the KFSI in DME-based electrolytes is superior than that of the KPF_6_ in EC/DEC- and diglyme-based electrolytes, indicating a better chemical stability with polysulfides of DME-based electrolytes, which could be attributed to the highly stable inorganic SEI layer formed on the surface of electrode by using KFSI in DME electrolytes [55, 56]. Therefore, we choose the KFSI in DME as electrolyte in this work.

The rate performances of the Co_0.85_Se-QDs/C-20, Co_0.85_Se-QDs/C-10, Co_0.85_Se-QDs/C-2, and Co_0.85_Se-QDs/C-0 were evaluated at various current densities from 50 to 1000 mA g^−1^. As shown in Fig. 4c, the Co_0.85_Se-QDs/C-20 electrode shows reversible capacities of 402, 369, 337, 296, and 253 mAh g^−1^ at constant current rates of 50, 100, 200, 500, and 1000 mA g^−1^, respectively. Even at the ultrahigh current density of 2000 mA g^−1^, it can still deliver a high reversible capacity of 220 mAh g^−1^, which can be considered as a quick discharge/charge electrode material. After 60 cycles at various current densities, the capacity is resumed to 401 mAh g^−1^ when the current density returns to 50 mA g^−1^, indicating good stability and rate performance. For most of the cycles, the capacity of the Co_0.85_Se-QDs/C-20 is relatively stable at each rate and the Coulombic efficiency is above 99%. However, the capacities of the Co_0.85_Se-QDs/C-10, Co_0.85_Se-QDs/C-2, and Co_0.85_Se-QDs/C-0 decrease rapidly to 137, 86, and 45 mAh g^−1^ at the current density of 2000 mA g^−1^, respectively. When the current density is abruptly set back to 50 mA g^−1^, the capacities of Co_0.85_Se-QDs/C-2 and Co_0.85_Se-QDs/C-0 cannot return to their initial values, which is due to the impure phases and structural collapse. The galvanostatic discharge curves of the Co_0.85_Se-QDs/C-20 at different rates are shown in Fig. S17a. Remarkably, the potassiation plateau is steady, demonstrating high reversibility. The long-term cycling performance is further investigated by the extended charge and discharge experiments (Fig. 4e). The original polyhedron structure of the Co_0.85_Se-QDs/C-20 is still retained after 100 cycles, indicating the stable potassium storage performance (Fig. S17b). At a current density of 1 A g^−1^, the Co_0.85_Se-QDs/C-20 electrode delivers a high initial reversible capacity of 280 mAh g^−1^ and stabilizes at around 228 mAh g^−1^ after 500 cycles. The excellent rate performance and long-term cycling performance could be ascribed to the high electronic conductivity and the stable hierarchical structure. In addition, a comparison between the electronic conductivities of the Co_0.85_Se-QDs/C-20 and pure Co_0.85_Se was made out by four-probe method [57] (Table S3), and the Co_0.85_Se-QDs/C-20 shows a high electronic conductivity.

The electrochemical dynamics of the Co_0.85_Se-QDs/C-20 electrode with potential pseudocapacitive behavior is studied. The CV curves at various scan rates from 0.1 to 2 mV s^−1^ are shown in Fig. S18a, displaying similar shapes with broad peaks during both cathodic and anodic processes. Based on the previous reports [58], the peak current (*i*) and the scan rate (*υ*) can follow the relationship of *i *=* av*^*b*^, where *i* is peak current, *ν* is scan rate and *a* and *b* are adjustable constants. Meanwhile, the *b* value can be obtained by the slope of the log (*i*) vs. log (*υ*) plot. If *b* value is close to 0.5, the charge storage is dominated by the ionic diffusion, whereas the *b* value of 1 represents a capacitive response [59, 60]. The *b* value of 0.77 for the Co_0.85_Se-QDs/C-20 electrode (Fig. S18b) indicates that the charge storage behavior is controlled by both the K-ion diffusion and the pseudocapacitive effect. Furthermore, the ratio of pseudocapacitive contribution can be further quantitatively determined by separating the equation of *i *=* k*_*1*_*v *+* k*_*2*_*v*^*1/2*^, where *k*_*1*_*v* and *k*_*2*_*v*^*1/2*^ represent the contribution of pseudocapacitance and ionic diffusion, respectively. Figure S18c shows the capacitive contributions of the Co_0.85_Se-QDs/C-20 electrode at 0.1, 0.2, 0.5, 1, and 2 mV s^−1^. The capacitive charge contribution rises with the increasing scanning rate. The capacitive-controlled capacity makes up about 47.3% in the total charge storage at 0.1 mV s^−1^ (Fig. S18d), which indicates the significant role of the capacitive charge storage in the total capacity. These results show that the charge storage mechanism contains the ion diffusion and the additional pseudocapacitance contribution, leading to a high capacity and rate capability.

The electrochemical impedance spectroscopy (EIS) was carried out to further research the electrochemical reaction process, and the corresponding Nyquist plots of the Co_0.85_Se-QDs/C-20, Co_0.85_Se-QDs/C-10, Co_0.85_Se-QDs/C-2, and Co_0.85_Se-QDs/C-0 electrodes in original cycle were measured (Fig. S19a). An equivalent circuit was used for fitting the electrochemical impedance spectra (insert of Fig. S19a). All of impedance spectra consist of a depressed semicircle in the high frequency range and a slope line in the low frequency range. The depressed semicircle corresponds to the electrode resistance (*R*_s_) and the charge transfer impedance (*R*_ct_), and the slope line is related to the Warburg impedance (*W*). For the original state, the charge transfer impedance of Co_0.85_Se-QDs/C-20 is lower than the other electrodes, portending the good ionic conductivity. The slope line in the low frequency range is almost vertical to the real axis in the imaginary part of the impedance, indicating its capacitor characteristic. The Nyquist plots of the Co_0.85_Se-QDs/C-20 electrode for different cycles are shown in Fig. S19b. The radius of the charge transfer part increases slightly in the subsequent cycle, indicating good reversibility.

The reasons for the excellent electrochemical performance of the as-prepared Co_0.85_Se-QDs/C-20 are as follows: (i) The tertiary hierarchical structure can reduce the K-intercalation stress in particle dimensions, provide large surface area, and prevent the agglomeration of active materials, facilitating the transportation of potassium ion and electron and maintaining the structural integrity. (ii) Because of the metallic property, the transportation of electrons in Co_0.85_Se is faster than other insulating anode materials. In addition, the presence of carbon skeleton can effectively enhance the electric conductivity of the composite, improving the rate capability. (iii) The nonignorable capacitive contribution has a significant influence on its high capacity and rate performance. These synergistic effects endow the Co_0.85_Se-QDs/C-20 with ultrahigh reversible capacity, excellent rate capability, and cycling stability. Here, we have to admit that the voltage plateau of K-ion full cell comprising Co_0.85_Se-QDs/C composite as an anode will be a bit lower than the classical graphite anode owing to the high delithiation potential of Co_0.85_Se-QDs/C composite. However, the cyclic stability, rate capability, and specific capacity of Co_0.85_Se-QDs/C composite are better than the classical graphite anode.

**Fig. 5:**
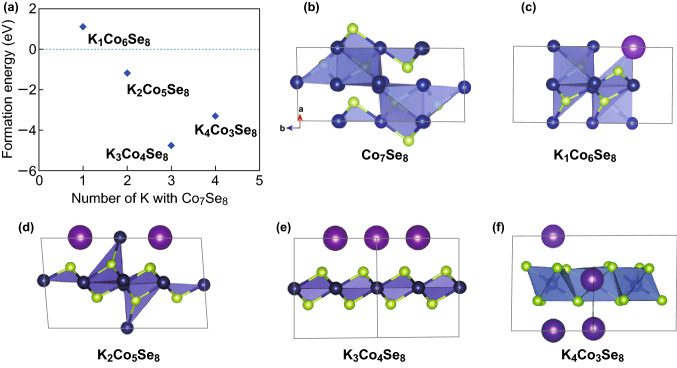
Density functional theory (DFT) calculations. **a** Relationship between the formation energy and the number of K. **b**–**f** Schematic molecular structures of the formed compounds

To further understand the potassium storage mechanism of the Co_0.85_Se, the first-principle calculations (Fig. 5) and ex situ XRD measurements (Fig. S20) were employed to investigate the replacement process of K ions into the Co_7_Se_8_ (closest to Co_0.85_Se) crystal structure. A positive formation energy (*E*_f_) indicates an endothermic and unstable K substitution reaction while a negative Ein suggests an exothermic and stable reaction. Figure 5 shows the calculated *E*_f_ with respect to the number of K ions reacting with Co_7_Se_8_ and schematic molecular structures. The corresponding structure information and total energy of different compositions are shown in Table S4. The replacement reactions for the Co_7_Se_8_ can be explained as follows:

5 $${\text{Co}}_{7} {\text{Se}}_{8} + {\text{ K}}^{ + } + {\text{ e}}^{ - } \to {\text{ K}}_{1} {\text{Co}}_{6} {\text{Se}}_{8} + {\text{ Co}},\quad E_{\text{f}} = \, 1.12\,{\text{eV}}$$

6 $${\text{Co}}_{7} {\text{Se}}_{8} + \, 2{\text{K}}^{ + } + \, 2{\text{e}}^{ - } \to {\text{ K}}_{2} {\text{Co}}_{5} {\text{Se}}_{8} + \, 2{\text{Co}},\quad E_{\text{f}} = \, - 1.18\,{\text{eV}}$$

7 $${\text{Co}}_{7} {\text{Se}}_{8} + \, 3{\text{K}}^{ + } + \, 3{\text{e}}^{ - } \to {\text{ K}}_{3} {\text{Co}}_{4} {\text{Se}}_{8} + \, 3{\text{Co}},\quad E_{\text{f}} = \, - 4.77\,{\text{eV}}$$

8 $${\text{Co}}_{7} {\text{Se}}_{8} + \, 4{\text{K}}^{ + } + \, 4{\text{e}}^{ - } \to {\text{ K}}_{4} {\text{Co}}_{3} {\text{Se}}_{8} + \, 4{\text{Co}},\quad E_{\text{f}} = \, - 3.32\,{\text{eV}}$$

It can be seen that the formation energies of reaction (6)–(8) are negative, suggesting that the three replacement reactions are favorable during cycling. Moreover, it is worth noting that reaction (7) exhibits the largest negative formation energy, suggesting that the substitution reactions for K ions from Co_7_Se_8_ into Co and K_3_Co_4_Se_8_ are energetically favorable. This spontaneous substitution reaction can well explain the high capacity of the Co_0.85_Se-QDs/C-20 electrode, which is similar to the conversion reaction of Na ions in CoSe_2_. We believe that the proposed strategy and results may offer a positive impression for the development of high-performance KIBs.

## Conclusions

In summary, we have successfully synthesized the Co_0.85_Se-QDs/C composite materials, which are used as an anode material for KIBs. This tertiary hierarchical structure together with the metallic nature of cobalt selenide QDs and their high mass loading and dispersity effectively achieves ultrahigh reversible capacity, good cycling stability, and excellent rate capability for high-performance KIBs. Co_0.85_Se-QDs/C-20 can deliver an ultrahigh capacity of 402 mAh g^−1^ at 50 mA g^−1^ after 100 cycles with the relatively high efficiency of approximately 99% by using the special KFSI in DME electrolyte. Even at a very high current density of 1 A g^−1^ over 500 cycles, the capacity can still maintain 228 mAh g^−1^. These results indicate that the Co_0.85_Se-QDs/C-20 could be a promising anode candidate for KIBs, and we expect that this synthetic route could guide the synthesis of edge-limited framework composites for energy storage and conversion.

## Electronic supplementary material

Below is the link to the electronic supplementary material.

Supplementary material 1 (PDF 2028 kb)
