# Supplementary material for: Tuning Metallic Co0.85Se Quantum Dots/Carbon Hollow Polyhedrons with Tertiary Hierarchical Structure for High-Performance Potassium Ion Batteries
Source: Nanomicro Lett. 2019 Nov 1;11:96. doi: 10.1007/s40820-019-0326-5 (PMC7770851; doi:10.1007/s40820-019-0326-5)
Supplement: Supplementary file 1 — Supplementary material 1 (PDF 2028 kb) [file 40820_2019_326_MOESM1_ESM.pdf]

Supporting Information for

Tuning Metallic $Co_{0.85}Se$ Quantum Dots/Carbon Hollow Polyhedrons with Tertiary Hierarchical Structure for High-Performance Potassium Ion Batteries

Zhiwei Liu[1], Kun Han[1], Ping Li[1, *], Wei (Alex) Wang[2, *], Donglin He[1], Qiwei Tan[1], Leying Wang[1], Yang Li[3], Mingli Qin[1], Xuanhui Qu[1]

[1]Beijing Advanced Innovation Center for Materials Genome Engineering, Institute for Advanced Materials and Technology, University of Science and Technology Beijing, Beijing 100083, People's Republic of China

[2]Beijing Key Laboratory of Bio-inspired Energy Materials and Devices, School of Space and Environment, Beihang University, Beijing 100191, People's Republic of China

[3]Department of Chemical Engineering, Polytechnique Montreal, Montreal, Quebec, H3C 3A7, Canada

Zhiwei Liu and Kun Han contributed equally to this work

*Corresponding authors. E-mail: ustbliping@126.com (Ping Li); wwang3@g.harvard.edu (Wei (Alex) Wang)

Supplementary Figures, Tables, and Discussion

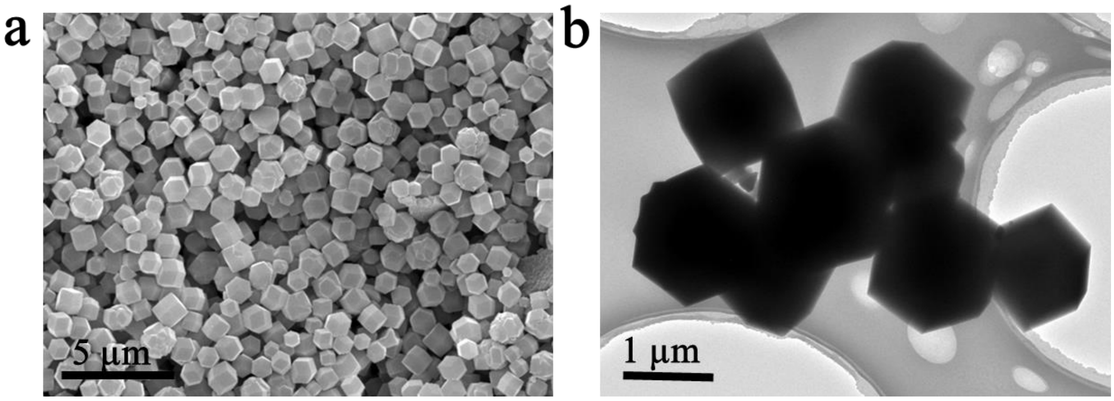

Fig. S1 a FESEM and **b** TEM images of ZIF-67

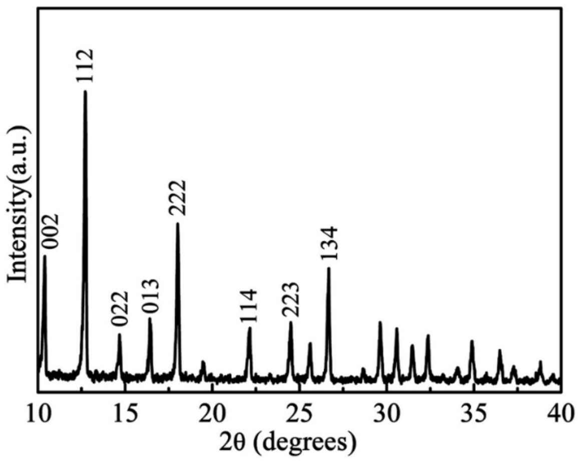

Fig. S2 XRD pattern of ZIF-67

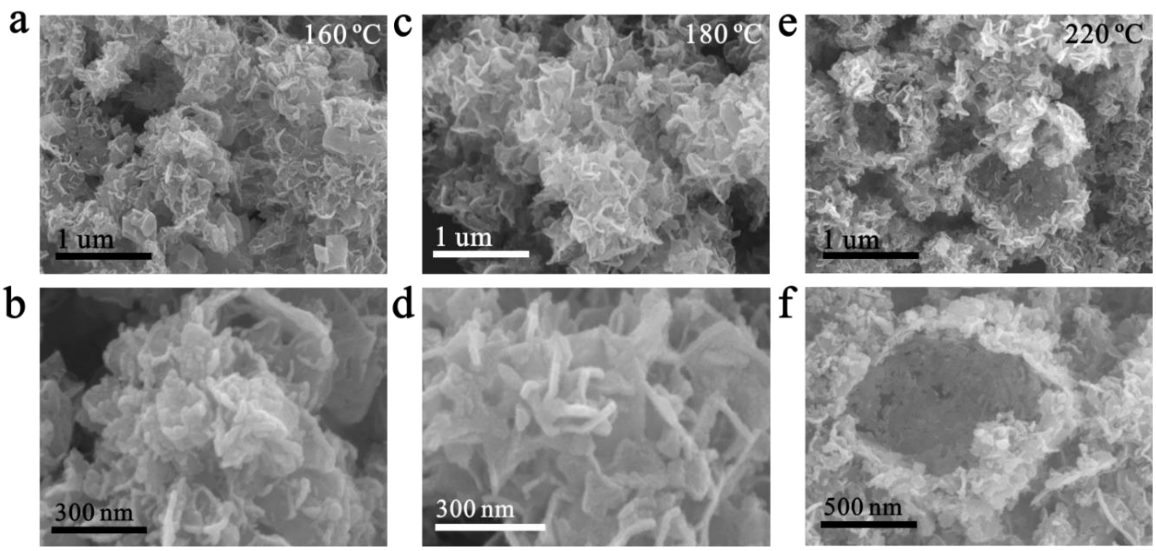

Fig. S3 FESEM images of **a, b** $Co_{0.85}Se$-QDs/C-160, **c, d** $Co_{0.85}Se$-QDs/C-180, and **e, f** $Co_{0.85}Se$-QDs/C-220

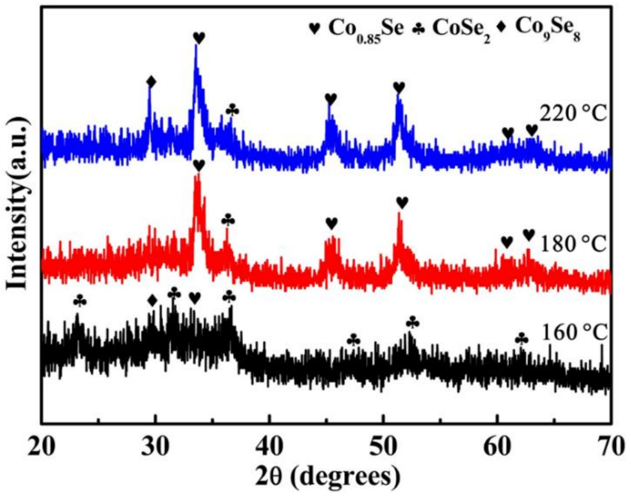

Fig. S4 XRD patterns of $Co_{0.85}Se$-QDs/C-160, $Co_{0.85}Se$-QDs/C-180, and $Co_{0.85}Se$-QDs/C-220

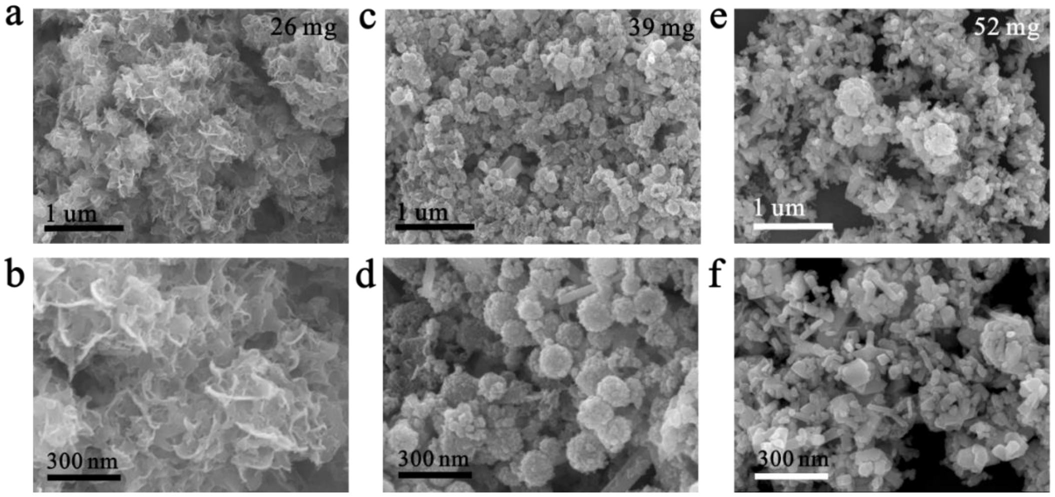

Fig. S5 FESEM images of **a, b** $Co_{0.85}Se$-QDs/C-26, **c, d** $Co_{0.85}Se$-QDs/C-39, and **e, f** $Co_{0.85}Se$-QDs/C-52

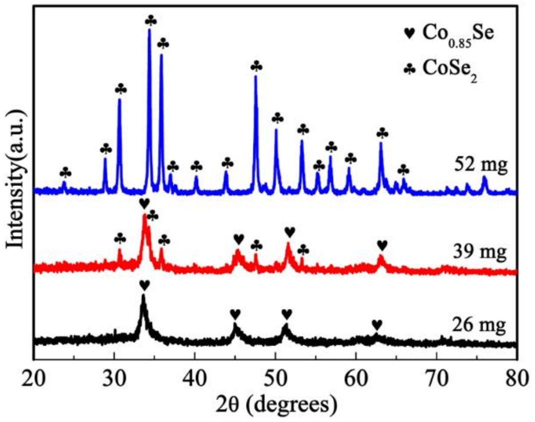

Fig. S6 XRD patterns of $Co_{0.85}Se$-QDs/C-26, $Co_{0.85}Se$-QDs/C-39, and $Co_{0.85}Se$-QDs/C-52

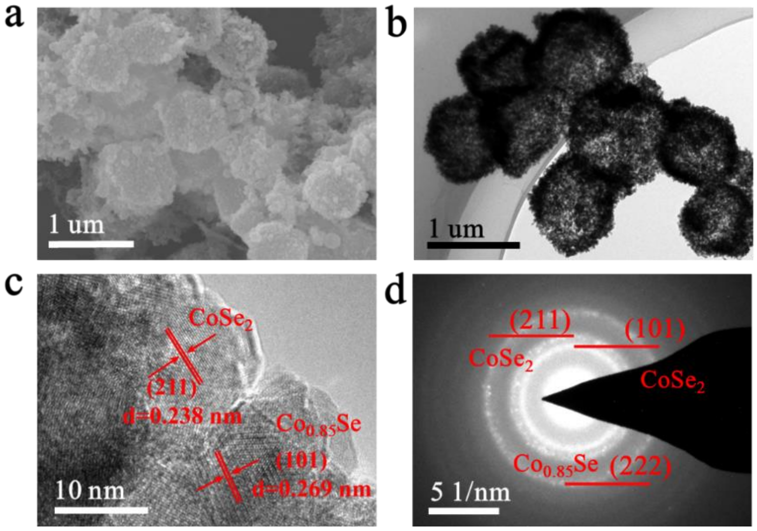

Fig. S7 a FESEM image, **b** TEM images, **c** HRTEM, and **d** SEAD images of $Co_{0.85}Se$-QDs/C-10

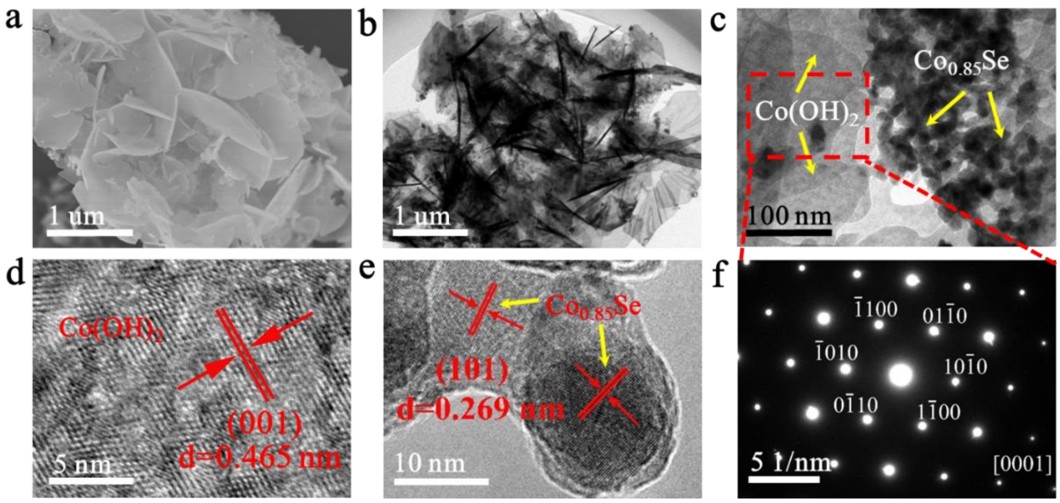

Fig. S8 a FESEM image, **b, c** TEM images, **d, e** HRTEM, and **d** SEAD images of $Co_{0.85}Se$-QDs/C-2

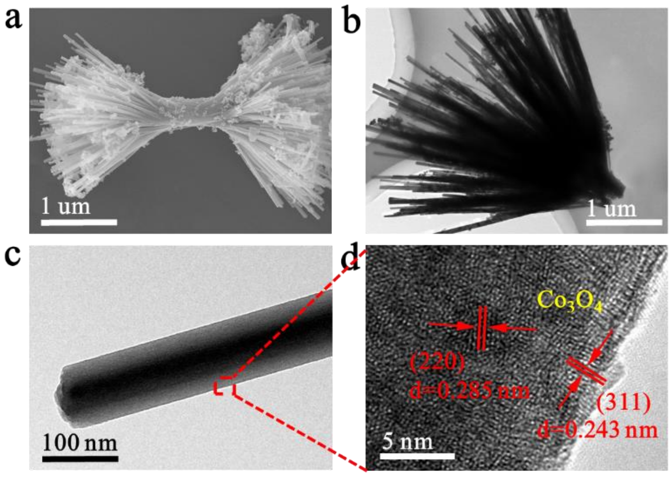

Fig. S9 a FESEM image, **b** TEM image, **c** HRTEM, and **d** SEAD images of $Co_{0.85}Se$-QDs/C-0

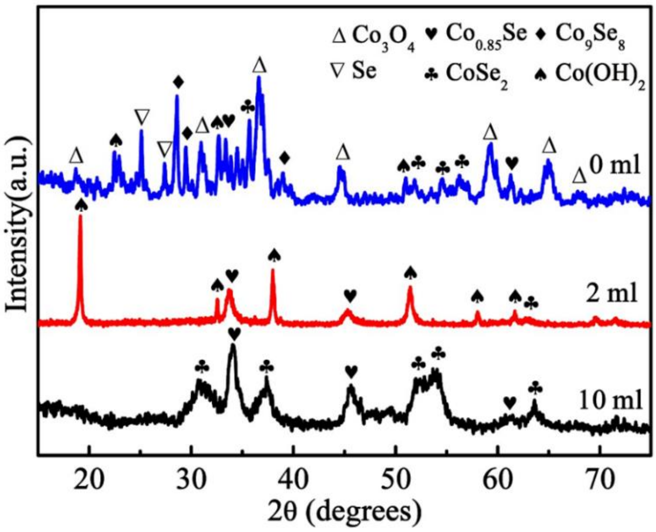

Fig. S10 XRD patterns of $Co_{0.85}Se$-QDs/C-10, $Co_{0.85}Se$-QDs/C-2 and $Co_{0.85}Se$-QDs/C-0

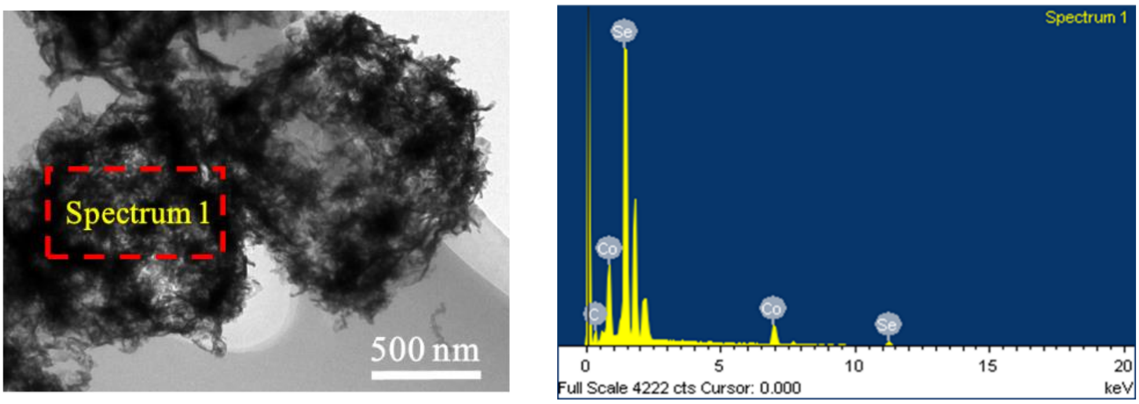

Fig. S11 TEM micrographs and EDS spectra of $Co_{0.85}Se$-QDs/C-20. Elemental spectra correspond to the Spectrum 1 point on the TEM micrograph

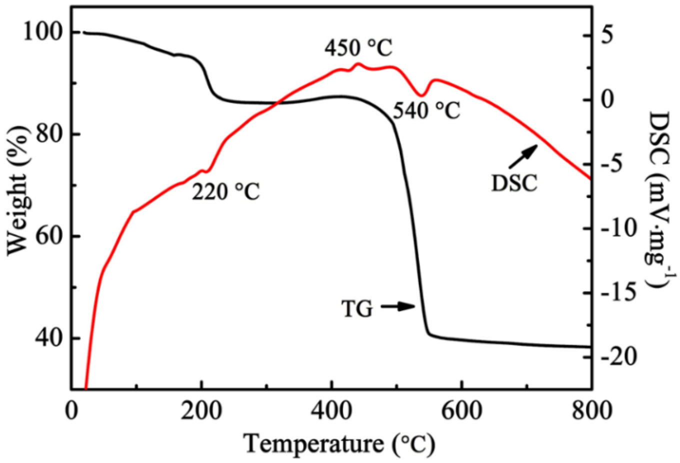

Fig. S12 TG-DSC curves of as-prepared $Co_{0.85}Se$-QDs/C-20

Figure S12 shows the TG/DSC curves of $Co_{0.85}Se$-QDs/C-20. A weight loss happened from 20 to 150 °C is attributed to the removal of the adsorbed water on the surfaces of $Co_{0.85}Se$-QDs/C-20. The weight loss in the range of 150 to 250 °C is contributed to the combustion of organic species in $Co_{0.85}Se$-QDs/C-20 [S1], corresponding to a small decalescence peak located at about 220 °C in the DSC curve. The weight increase occurred at 400 °C due to the partial oxidation of $Co_{0.85}Se$ to $CoSeO_4$ and SeO_2, and subsequent acute weight losses occurred in the range of 500 to 550 °C due to the oxidation of $Co_{0.85}Se$ into Co_3O_4, the conversion of $CoSeO_4$ to Co_3O_4 and the evaporation of SeO_2 [S2]. In addition, the complex reactions are accompanied with the oxidation of carbon. The weight decrease corresponds to an obvious decalescence peak at 540 °C. The TG result also confirmed the mass content of carbon in the $Co_{0.85}Se$-QDs/C-20. The total reaction can be simply written as:

$$3Co_{0.85}Se_{(S)} + 4.7O_{2\,(g)} \rightarrow 0.85Co_3O_{4\,(S)} + 3SeO_{2\,(g)} \qquad (S1)$$

$$C_{(s)} + O_{2\,(g)} \rightarrow CO_{2\,(g)} \qquad (S2)$$

According to the final weight of the Co_3O_4, it can be calculated the weight of the $Co_{0.85}Se$ and

carbon. The original weight is kept for the final products is 38%. Except for the common weight loss of adsorption water and organic species of 13%, based on the reaction (S1), the mass content of $Co_{0.85}Se$ and carbon in $Co_{0.85}Se$-QDs/C-20 is calculated as about 72 wt% (0.38×3×129.15/(0.85×241) = 72 wt%) and 15 wt%, respectively.

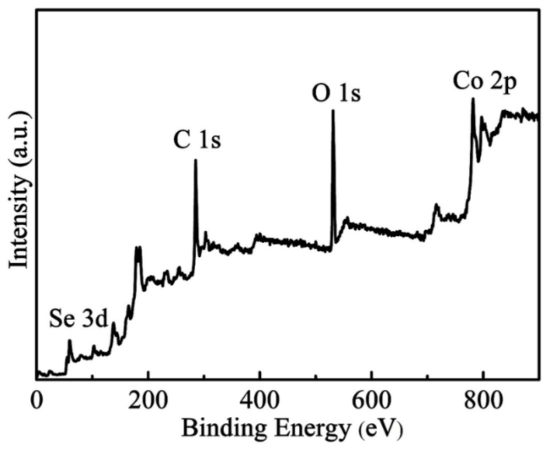

Fig. S13 The survey XPS spectrum of as-prepared $Co_{0.85}Se$-QDs/C-20

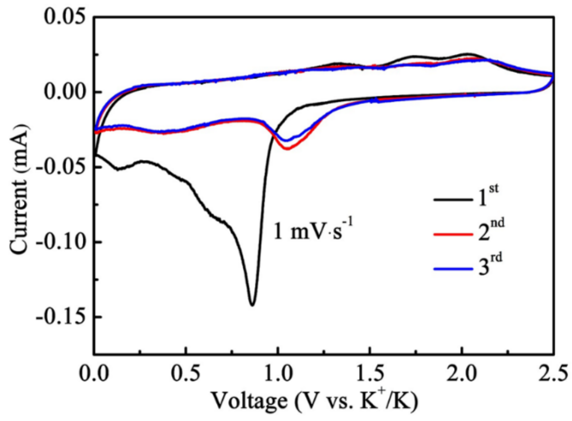

Fig. S14 Cyclic voltammograms of $Co_{0.85}Se$-QDs/C-20 in the voltage range of 0.01-2.5 V at a scan rate of 0.1 mV s^{-1}

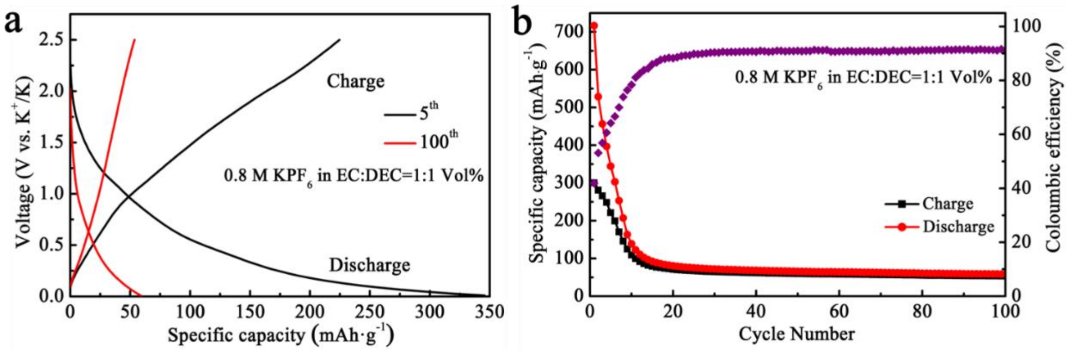

Fig. S15 a Discharge/charge voltage profiles of $Co_{0.85}Se$-QDs/C-20 with the 5th and 100th cycling. **b** Cycling performance of $Co_{0.85}Se$-QDs/C-20 at a current rate of 50 mA g^{-1} when using the electrolyte of 0.8 M KPF_6 in EC: DEC

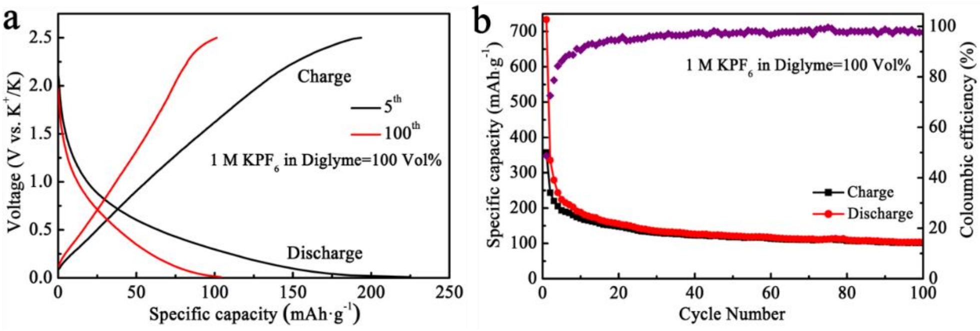

Fig. S16 a Discharge/charge voltage profiles of $Co_{0.85}Se$-QDs/C-20 with the 5th and 100th cycling. **b** Cycling performance of $Co_{0.85}Se$-QDs/C-20 at a current rate of 50 mA g^{-1} when using the electrolyte of 1 M KPF_6 in Diglyme

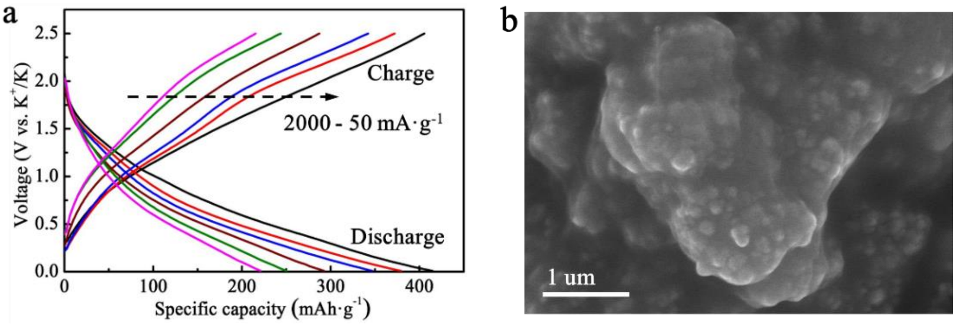

Fig. S17 a Galvanostatic discharge/charge curves of $Co_{0.85}Se$-QDs/C-20 at different rates. From right to left: 50, 100, 200, 500, 1000, and 2000 mA g^{-1}, **b** FESEM images of the $Co_{0.85}Se$-QDs/C-20 electrode after 100 cycles at a current density of 100 mA g^{-1}

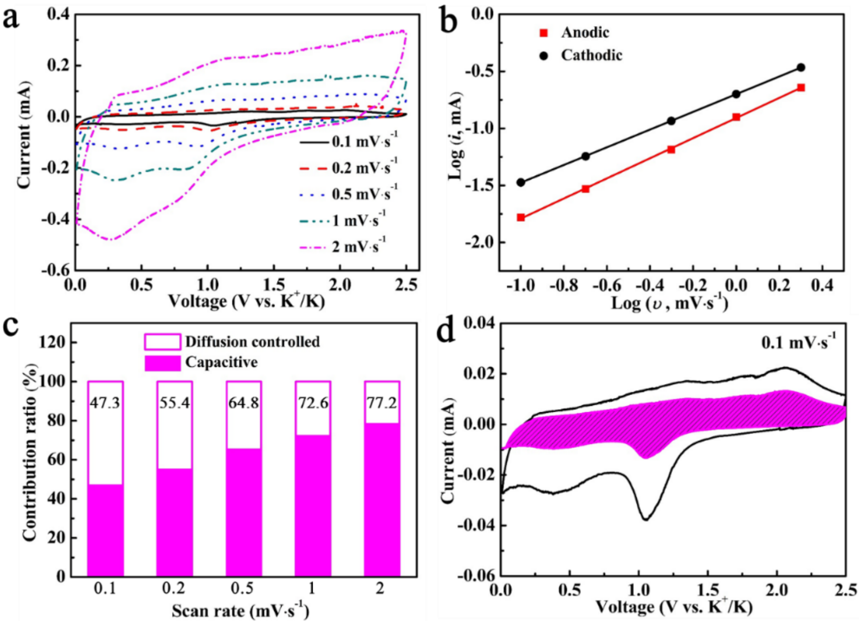

Fig. S18 The potential pseudocapacitive behavior of the $Co_{0.85}Se$-QDs/C-20 electrode. **a** CV profiles at different scan rates. **b** the plots of log(i) vs. log(υ) (peak current: i, scan rate: υ) of $Co_{0.85}Se$-QDs/C-20. **c** The percentages of pseudocapacitive contributions at different scan rates. **d** The purple region shows the CV profile with the pseudocapacitive contribution at scan rate of 0.1 mV s^{-1}

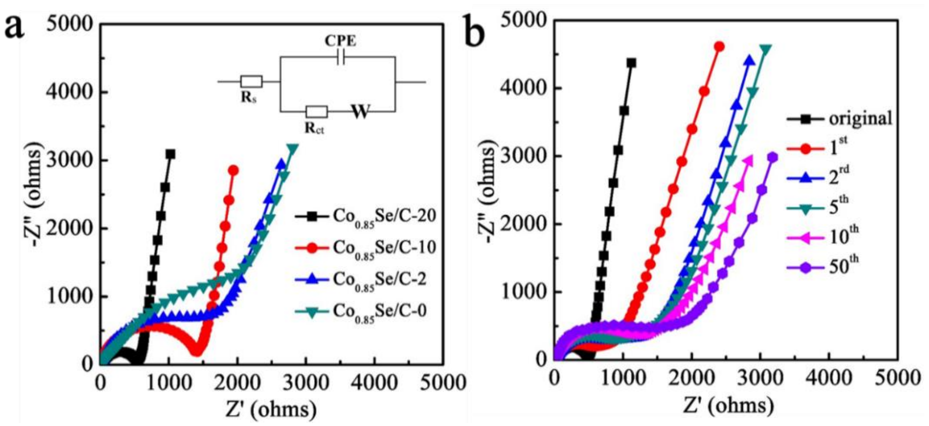

Fig. S19 a Nyquist plots of $Co_{0.85}Se$-QDs/C-20, $Co_{0.85}Se$-QDs/C-10, $Co_{0.85}Se$-QDs/C-2, and $Co_{0.85}Se$-QDs/C-0 in original cycle (insert of the simplified equivalent circuit model). **b** Nyquist plots of $Co_{0.85}Se$-QDs/C-20 in the original, 1st, 2nd, 5th, 10th, and 50th cycling cycles

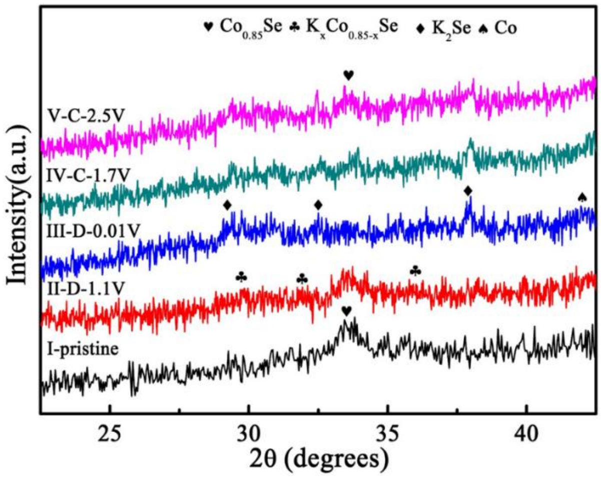

Fig. S20 The *ex-situ* XRD patterns of $Co_{0.85}Se$-QDs/C-20 electrode at various voltage

During the first discharge cycle, the formation of $K_xCo_{0.85-x}Se$ by insertion of the K-ion into the interlayer of $Co_{0.85}Se$ is similar to the reaction process of $Co_{0.85}Se$ in LIBs [S3]. At the discharge voltage from 1.1 to 0.01 V, the appearance of K_2Se peaks was attributed to the conversion reaction of $K_xCo_{0.85-x}Se$ to form K_2Se and metal Co. When the charge voltage returns to 2.5 V, the obvious $Co_{0.85}Se$ peaks show that K_2Se and metal Co are converted back to $Co_{0.85}Se$.

Table S1 The element content of $Co_{0.85}Se$-QDs/C-20 via EDS analysis

Element	Weight (%)	Atomic (%)
C	27.75	69.05
Co	28.45	14.40
Se	43.80	16.55
Totals	100.00	100.00

Table S2 Electrochemical performance comparison of some reported negative materials of KIBs

Active materials	Electrochemical performance	References
**$Co_{0.85}Se$-QDs/C-20**	**402 mAh g^{-1} for 100 cycles at 50 mA g^{-1}, 228 mAh g^{-1} for 500 cycles at 1A g^{-1}**	**This work**
Graphite	100 mAh g^{-1} for 50 cycles at 140 mA g^{-1}	[S4]
Soft carbon	185 mAh g^{-1} for 50 cycles at 558 mA g^{-1}	[S4]
Hard carbon	216 mAh g^{-1} for 100 cycles at 27.9 mA g^{-1}	[S5]
S, O-codoped hard carbon	201 mAh g^{-1} for 200 cycles at 200 mA g^{-1}	[S6]
Carbon nanofibers	80 mAh g^{-1} after 20 cycles at 50 mA g^{-1}	[S7]
NCNFs-650	248 mAh g^{-1} after 100 cycles at 500 mA g^{-1}	[S8]
RGO films	120 mAh g^{-1} after 100 cycles at 10 mA g^{-1}	[S9]
Polynanocrystalline graphite	80 mAh g^{-1} after 300 cycles at 100 mA g^{-1}	[S10]
N-doped graphene	210 mAh g^{-1} after 100 cycles at 100 mA g^{-1}	[S11]
VSe_2 nanosheet	150 mAh g^{-1} after 500 cycles at 2 A g^{-1}	[S12]
N, P-codoped carbon/CoP	127 mAh g^{-1} after 1000 cycles at 100 mA g^{-1}	[S13]
$MoSe_2$/C	322 mAh g^{-1} after 100 cycles at 200 mA g^{-1}	[S14]
MoS_2@SnO_2@C	312 mAh g^{-1} after 25 cycles at 50 mA g^{-1}	[S15]
$KTi_2(PO_4)_3$@C	221 mAh g^{-1} after 50 cycles at 20 mA g^{-1}	[S16]
MoS_2	65 mAh g^{-1} after 200 cycles at 20 mA g^{-1}	[S17]
a-Ti_3C_2	98 mAh g-1 for 20 cycles at 20 mA g-1	[S18]

Table S3 A comparison of the electronic conductivity of the $Co_{0.85}Se$-QDs/C-20 and pure $Co_{0.85}Se$

**Sample**	**Electronic conductivity (S m^{-1})**
$Co_{0.85}Se$-QDs/C-20	2.4×10^{3}
$Co_{0.85}Se$	2.9×10^{-5}

Table S4 The structure information and total energy of different compositions

**Sample**	**a**	**b**	**c**	**α**	**β**	**γ**	**Volume**	**Total energy (eV)**
**Co_7Se_8**	6.274	7.186	7.189	74	60	106	231.46	-80.77
**Co**	2.530	2.530	3.994	90	90	120	22.14	-14.31
**K**	5.259	5.259	5.259	90	90	90	145.44	-2.28
**$K_1Co_6Se_8$**	6.773	7.300	7.146	76	60	104	270.80	-73.64
**$K_2Co_5Se_8$**	7.813	7.164	7.166	73	61	100	310.30	-69.92
**$K_3Co_4Se_8$**	8.327	7.288	7.130	78	61	103	339.94	-67.49
**$K_4Co_3Se_8$**	8.436	7.765	7.436	77	61	103	382.78	-60.03

Supplementary References

[S1] W. Ma, Y.F. Guo, X.H. Liu, D. Zhang, T. Liu et al., Nickel dichalcogenide hollow spheres: controllable fabrication, structural modification, and magnetic properties. Chem. Eur. J. **19,** 15467-15471 (2013). https://doi.org/10.1002/chem.201302716

[S2] Z.P. Li, H.T. Xue, J.Q. Wang, Y.B. Tang, C.S. Lee, S.R. Yang, Reduced graphene oxide/marcasite-type cobalt selenide nanocrystals as an anode for lithium-ion batteries with excellent cyclic performance. ChemElectroChem **2**, 1682-1686 (2015). https://doi.org/10.1002/celc.201500179

[S3] J.S. Zhou, Y. Wang, J. Zhang, T.P. Chen, H.H. Song, H.Y. Yang, Two dimensional layered $Co_{0.85}Se$ nanosheets as a high-capacity anode for lithium-ion batteries. Nanoscale **8**, 14992-15000 (2016). https://xs.scihub.ltd/10.1039/C6NR03571J

[S4] Y.F. Dong, Z.S. Wu, S.H. Zheng, X.H. Wang, J.Q. Qin et al., Ti_3C_2 MXene-derived sodium/potassium titanate nanoribbons for high-performance sodium/potassium ion batteries with enhanced capacities. ACS Nano **11**, 4792-4800 (2017). https://doi.org/10.1021/acsnano.7b01165

[S5] B.F. Ji, F. Zhang, X.H. Song, Y.B. Tang, A novel potassium-ion-based dual-ion battery. Adv. Mater. **29**, 1700519 (2017). https://doi.org/10.1002/adma.201700519

[S6] M. Chen, W. Wang, X. Liang, S. Gong, J. Liu, Q. Wang, S.J. Guo, H. Yang, Sulfur/oxygen codoped porous hard carbon microspheres for high-performance potassium-ion batteries. Adv.

Energy Mater. **8**, 1800171 (2018). https://doi.org/10.1002/aenm.201800171

[S7] Z.L. Jian, Z.Y. Xing, C. Bommier, Z.F. Li, X.L. Ji, Hard carbon microspheres: potassium-ion anode versus sodium-ion anode. Adv. Energy Mater. **6**, 1501874 (2016). https://doi.org/10.1002/aenm.201501874

[S8] Y. Xu, C.L. Zhang, M. Zhou, Q. Fu, C.X. Zhao, M.H. Wu, Y. Lei, Highly nitrogen doped carbon nanofibers with superior rate capability and cyclability for potassium ion batteries. Nat. Commun. **9**, 1720 (2018). https://doi.org/10.1038/s41467-018-04190-z

[S9] J. Han, M.W. Xu, Y.B. Niu, G.N. Li, M.Q. Wang et al., Exploration of $K_2Ti_8O_{17}$ as an anode material for potassium-ion batteries. Chem. Commun. **52**, 11274-11276 (2016). https://doi.org/10.1039/C6CC05102B

[S10] J. Han, Y.B. Niu, S.J. Bao, Y.N. Yu, S.Y. Lu, M.W. Xu, Nanocubic $KTi_2(PO_4)_3$ electrodes for potassium-ion batteries. Chem. Commun. **52**, 11661-11664 (2016). https://doi.org/10.1039/C6CC06177J

[S11] K. Share, A.P. Cohn, R. Carter, B. Rogers, C.L. Pint, Role of nitrogen-doped graphene for improved high-capacity potassium ion battery anodes. ACS Nano **10**, 9738-9744 (2016). https://doi.org/10.1021/acsnano.6b05998

[S12] C. Yang, J.R. Feng, F. Lv, J.H. Zhou, C.F. Lin et al., Metallic graphene-like VSe_2 ultrathin nanosheets: superior potassium-ion storage and their working mechanism. Adv. Mater. **30**, 1800036 (2018). https://doi.org/10.1002/adma.201800036

[S13] J. Bai, B.J. Xi, H.Z. Mao, Y. Lin, X.J. Ma, J.K. Feng, S.L. Xiong, One-step construction of N,P-Co-doped porous carbon sheets/CoP hybrids with enhanced lithium and potassium storage. Adv. Mater. **30**, 1802310 (2018). https://doi.org/10.1002/adma.201802310

[S14] W. Wang, B. Jiang, C. Qian, F. Lv, J.R. Feng et al., Pistachio-shuck-like $MoSe_2$/C core/shell nanostructures for high-performance potassium-ion storage. Adv. Mater. **30**, 1801812 (2018). https://doi.org/10.1002/adma.201801812

[S15] Z. Chen, D.G. Yin, M. Zhang, Sandwich-like MoS_2@SnO_2@C with high capacity and stability for sodium/potassium ion batteries. Small **14**, 1703818 (2018). https://doi.org/10.1002/smll.201703818

[S16] Z.X. Wei, D.X. Wang, M.L. Li, Y. Gao, C.Z. Wang, G. Chen, F. Du, Fabrication of hierarchical potassium titanium phosphate spheroids: a host material for sodium-ion and potassium-ion storage. Adv. Energy Mater. **8**, 1801102 (2018). https://doi.org/10.1002/aenm.201801102

[S17] X.D. Ren, Q. Zhao, W.D. McCulloch, Y.Y. Wu, MoS_2 as a long-life host material for potassium ion intercalation. Nano Res. **10**, 1313-1321 (2017). https://doi.org/10.1007/s12274-016-1419-9

[S18] P.C. Lian, Y.F. Dong, Z.S. Wu, S.H. Zheng, X.H. Wang et al., Alkalized Ti_3C_2 MXene nanoribbons with expanded interlayer spacing for high-capacity sodium and potassium ion batteries. Nano Energy **40**, 1-8 (2017). https://doi.org/10.1016/j.nanoen.2017.08.002
